# Supplementary material for: Are carbon emissions trading and green financial instruments synergistic? -Comprehensive quantitative research based on content analysis
Source: PLoS One. 2024 Mar 7;19(3):e0298601. doi: 10.1371/journal.pone.0298601 (PMC10919601; doi:10.1371/journal.pone.0298601)
Supplement: S1 Data — (ZIP) [file pone.0298601.s001.zip › Data/地级市Z_F工作报告.zip!/╝¬┴╓/═¿╗»╩╨/└┤╫╘╣1⁄2╓┌║┼í╛┬φ┐╦╩2╛▌═°í┐_2011.docx]

一、“十一五”和2010年工作回顾

　　“十一五”是我市经历重大挑战、取得重大成就的五年。在省委、省政府和市委的坚强领导下，在市人大、市政协的监督支持下，我们紧紧依靠和带领全市人民，咬定发展不放松，积极应对复杂多变的国内外经济环境特别是国际金融危机严重冲击，妥善处置涉及发展稳定的大事难事，全力推进通化老工业基地振兴，“十一五”规划确定的目标任务全面完成，经济发展、社会进步、民生改善、和谐稳定普结硕果。

　　经济持续快速增长。预计：2010年实现地区生产总值600亿元，是2005年的2.6倍，年均增长18%；全口径财政收入61.7亿元，是2005年的2.6倍，年均增长20.7%，其中地方级财政收入34亿元，是2005年的3.7倍，年均增长29.9%；规模以上工业总产值895亿元，是2005年的3.8倍，年均增长30.6%；社会消费品零售总额249.4亿元，是2005年的2.7倍，年均增长21.8%；外贸进出口总额6.2亿美元，是2005年的2.3倍，年均增长18.2%。节能减排完成了省下达任务。

　　项目建设成效显著。连续五年开展了“项目建设年”活动，固定资产投资累计完成2300亿元，比“十五”时期净增2006亿元，年均增长48.4%。实施3000万元以上项目1878项、亿元以上项目416项，工业项目投资比重达到50.4%。100万吨冷轧、40万吨硅钢、3000公斤人胰岛素、45万吨特优级食用酒精、20万吨啤酒、500千伏输变电、200兆瓦热电联产等301项亿元以上项目建成投产。通沈高速公路、市区绕越线暨单塔斜拉桥竣工通车。建成高速公路118公里，新增干线公路509公里，新建农村公路3568公里。积极争取扩大内需政策支持，396个项目列入国家投资计划，争取资金26.6亿元。

　　结构调整取得突破性进展。三次产业比例由16.1：46.3：37.6调整到11：52.8：36.2。粮食产量达到35亿斤阶段性水平，畜牧业和园艺特产业产值年均分别增长15%和8%，农产品加工量达到200万吨。规模以上工业企业发展到694户、新增412户。医药、冶金、食品三大支柱产业产值分别达到300亿元、240亿元、135亿元，是2005年的4.1倍、2.2倍、7.6倍。能源、新型建材、机械制造等优势产业实现较快发展。技改投资完成659亿元，新产品实现产值365.5亿元，分别是“十五”的6.6倍、4.6倍。国家高新技术企业发展到18户。新创中国驰名商标5件。第三产业增加值年均增长16.7%。欧亚购物中心、义乌国际商贸城等一批重点商贸项目建成运营。实施旅游建设项目120项，旅游收入是2005年的3.7倍。民营经济主营业务收入年均增长26.3%，实缴税金年均增长33.1%。

　　改革开放迈出新步伐。完成了新一轮政府机构改革。调整理顺了市对区的财政管理体制。妥善解决了150多户改制企业遗留问题。土地流转、粮食流通、农业保险等农村改革取得新成效。集体林权制度主体改革顺利完成，70.8万公顷林地确权到户。收储土地1768公顷，招拍挂出让1282公顷。吉林银行通化分行挂牌营业，小额贷款公司运营28家。新增上市公司3户。招商引资到位资金2175亿元，引进千万元以上项目5000项，新批外商投资企业46户，香港华润、江苏高力等一批知名企业落户通化。通化经济开发区和7个县（市、区）开发区基本形成规模。

　　城乡面貌发生较大变化。市区累计投入资金131亿元，实施了50多项城建工程。拆迁棚户区310万平方米，开工建筑面积565万平方米。集中供热规模达到1280万平方米。解决了城区周边12.9万人吃水和2.3万户居民用气问题。完成了玉带河改造工程。新增绿地172万平方米，建设和改造了155条主次街路和倚江园、沿江景观带、玉皇山公园等9个广场、公园。提升了滨江沿河、城区街路的亮化水平，路灯总数超过3.5万盏。江南新区有序开发，城市空间框架已经拉开。实施了一批污染治理和生态环保工程，市区空气质量优良天数达到315天。启动新农村建设项目8481项，全面实施“清洁文明”工程，农村环境综合整治取得新成效。修建主要江河堤防65公里。植树造林150万亩、封山育林140万亩，实现了连续56年无重大森林火灾。

　　社会事业蓬勃发展。承担国家和省级科研项目178项，建成省级以上技术研发中心27家，授权专利899项。基础教育、职业教育继续发展，新建和改造校舍77万平方米，中小学普及现代信息技术教育，组建了通化技师学院，完成了职教园区一期工程。艺术创作获国家和省级奖项68项，农家书屋覆盖率达到100%，文物保护和非物质文化遗产申报工作全面加强。医药卫生体制改革继续深化，公共卫生和医疗服务保障体系进一步完善，新建、改扩建医疗卫生机构104个、30万平方米，各类传染病得到有效控制，医疗服务和医疗救治水平明显提高。群众体育活动广泛开展，竞技体育获省级以上金牌410枚。广播电视、计划生育、妇女儿童、老龄、残疾人等事业长足发展。社区建设和管理工作进一步加强。民族、宗教、外事、侨务、人防、气象、地震监测和军民共建等工作都取得了新成绩。文明和谐创建活动深入推进，城乡文明程度显著提高。

　　人民生活进一步改善。城镇居民人均可支配收入由“十五”期末的8807元提高到16700元，年均增长13.7%；农民人均纯收入由3327元提高到6152元，年均增长13.1%；职工平均工资由12185元提高到22000元，年均增长12.5%。城镇、农村居民人均住房面积分别增至28.5和23.5平方米。住房公积金缴存年均增长36%。城镇新增就业29.4万人，新增社会保险参保103.3万人。企业退休人员养老金提高到1127元，接近翻一番。城乡低保实现应保尽保。新型农村合作医疗参合率达到98.7%，百万农民看病就医得到基本保障。建立了覆盖城乡的社会救助体系，年均救助困难群众16.7万人。实施“六路安居”工程，改善了13.1万户困难群众住房条件。建成农村饮水安全工程1011处，55.7万农村人口受益。

　　“十一五”期间，我市固定资产投资增速和驰名商标、国家地理标志保护品种、企业上市数量位列全省前茅，获得中国人居环境奖，被命名为中国通化葡萄酒城、中国医药城、中国特色魅力城市、中国百佳投资城市、中国旅游竞争力百强城市、中国十佳投资创业城市、中国十佳休闲宜居生态城市、中国最具人文情怀城市、中国最具幸福感城市，被认定为生物产业国家高技术产业基地、首批国家新型工业化医药产业示范基地、中国人参产业基地、中国松花砚之乡、首批低碳国土实验区，荣获全国双拥模范城“六连冠”、全国质量兴市先进市等殊荣。

　　2010年是“十一五”的最后一年。全市上下攻坚克难，保持经济社会又好又快发展。地区生产总值增长15%，全口径财政收入增长30.7%，地方级财政收入增长30.8%，固定资产投资完成800亿元、增长37.6%，主要指标增速在全省排序靠前。

　　实施投资拉动、项目带动和创新驱动战略，发展活力持续增强。在建和新建项目数量分别增长22.1%、26.4%，项目数量和质量创历史最好水平。谋划启动了“千亿级”生物产业基地和千万吨级新型钢铁基地两个结构调整项目，5万吨金属镁合金及深加工、12万吨家电彩涂板、长白山医药物流中心、人参交易中心等203项超亿元项目相继开工，百万吨熟料生产线等重点项目竣工投产。中东新天地购物公园、恒坤家居博览购物中心建成运营。招商引资到位资金730亿元，引进千万元以上项目1600项，实际利用外资1.65亿美元。300万吨精品钢、湾湾川生态新城等大项目成功签约。保利、华能等大型央企进入通化。国家知识产权试点城市正式揭牌。通化医药高新技术产业开发区晋升为省级园区。通丹经济带列入省“十二五”规划。成功承办了东北东部（12+1）圆桌会议，区域合作、开发开放迈出实质性一步。

　　推动产业升级和企业发展，工业化水平稳步提高。规模以上工业增加值增长23.8%，利润增长1.4倍。实施工业产业跃升计划，医药、冶金、食品等八大产业产值增长30.1%。启动工业“五个百”工程，百个投资亿元以上项目完成投资135亿元，百个技术改造项目完成投资69亿元，百个投产达效项目实现产值67亿元，重点扶持的百户企业实现产值占规模以上工业的75%，新增规模以上工业企业107户。通钢与首钢实现重组，67个兴钢项目竣工投产。东宝、华夏等企业的域外合作重组成功实施。建立了出口食品和农产品质量安全示范区、省级钢材出口基地和果仁出口基地。国家石油机械产品质量检验中心投入运行。组建了企业融资和土地收储两个平台。争取国家开行、招商银行授信额度120亿元。双龙化工在深交所上市，金宝药业和万家福食品在天津股权交易所融资挂牌。

　　狠抓基础设施和城市建设，城镇化进程步伐加快。通丹、梅沈高速公路和通丹铁路建设快速推进，营抚高速公路竣工通车，通靖高速公路即将开工，通长高速公路可研上报交通部、国家发改委，通集高速公路通过省审批，通化机场开工，通化陆港奠基。改造市区棚户区及危旧房113万平方米。“暖房子”工程和城市环境综合整治成效明显，改造老旧楼237栋、陈旧管网80公里，撤并小锅炉31座，建筑节能和热计量改造100余万平方米，拆除仓房和危违建筑3911处。完成了集中供热三期工程，新增供热面积600万平方米。109条道路和3座桥面完成升级改造，跨江人行景观桥、滨江东路佐安河口至张家大桥段改造工程基本完工，新华大街等3条精品街和绿化美化亮化、江南公园等工程如期竣工。解决了3万人全天用水问题，新增煤气用户4000户，新建、维修公厕100座。完成了“十一五”环保建设项目，市区和梅河口市、辉南县、柳河县污水处理厂建成投入使用。市容环境卫生和市政公用设施及物业管理办法颁布实施，城市品位和功能进一步提升。出台了统筹推进城镇化实施意见，市县两级城市总体规划完成修编。通化被列为全国数字地理空间框架建设试点城市。

　　加快农村和县域经济发展，农业现代化建设扎实推进。粮食生产在大灾之年夺得丰收。新建标准化牧业小区146个。1007公顷现代农业园区龙头棚膜蔬菜建设项目启动。县域工业和农业产业化实现较快发展，中新工业园等一批项目开工，9个千万元以上农业产业化项目建成投产，市级以上龙头企业发展到155户，绿色有机无公害农产品标识达到252种，建成9个国家级农业标准化示范区。与中科院签署了人参产业战略合作框架协议。实施“百村示范、全面提升”工程，113个省级示范村基础设施、公益事业建设日益完善。推进乡（镇）“三资”代理服务中心和政务服务中心建设，村级“三项制度”得到落实。开展了“十佳乡镇”、“十佳魅力乡村”、“十佳创业致富带头人”评选活动。7个县（市、区）生产总值增长16%，全口径财政收入全部超过5亿元，其中梅河口市达到13.8亿元。在全省县域综合实力排序中，梅河口市位居第4位，通化县进入前10位、晋升4位，集安市和柳河县分别晋升3位，辉南县晋升1位。

　　坚持保障和改善民生，社会事业进一步发展。制定实施了民生工作计划，十个方面、百件实事普惠广大群众。企业退休人员养老金月均增加140元，城市低保月保障标准提高到260元，农村低保年保障标准提高到1410元，新型农村合作医疗政府补贴增至120元。3000多户城市低保边缘户纳入社会救助体系，为1.68万户城镇低收入家庭发放了住房补贴。提高了社区办公经费补贴标准和社区干部工资。改造农村危旧房6759户、工矿棚户区29万平方米。校舍安全工程完成三年任务的91.7%。完成了市青少年宫改建、精神病院扩建、儿童福利院建设工程。市医院扩建、中医院易地新建、医疗垃圾无害化处理、哈泥河污染治理等工程基本完工。电视剧《远去的飞鹰》完成拍摄，修缮了高志航纪念馆。《通化市志1986—2005》出版发行。建成体育健身广场144个。市科技馆、历史博物馆、自然博物馆、群众艺术馆、图书馆开工，关东文化产品交易市场、松花奇石展览中心建成开业，国际文化旅游教育产业园落户通化。全省首届松花石产业发展研讨会在我市召开。举办了元宵节灯会、雪花啤酒节等系列活动，节日文化、广场文化、社区文化丰富多彩。采取发展生产、保障供应、强化监管等措施，保证了蔬菜副食品供应和价格基本稳定。

　　坚决保卫人民群众生命财产安全，抗洪抢险救灾斗争取得重大胜利。去年，面对特大洪涝灾害，我们紧急行动，有力有序有效组织群众做好预防和紧急避险，及时排查、超前防御次生地质灾害，紧急转移安置受灾群众30万人次；在最短时间内恢复了供水、供电、通讯、交通等设施功能，妥善处置了市区自来水管线被冲断等突发事件；完成房屋倒塌重建、损坏修复2.8万户，入冬前受灾户全部搬进新居，保证了受灾群众有饭吃、有衣穿、有住处、有洁净饮用水、有病能得到医治。在这场艰苦卓绝的斗争中，全市党政军民以实际行动诠释了弥足珍贵的“以人为本、尊重科学，不惧艰险、顽强拼搏，勇于担当、无私奉献，众志成城、敢于胜利”的通化抗洪精神。

　　加强民主法制建设，社会管理和公共服务水平进一步提升。坚持重大事项向人大常委会报告、向政协通报制度，认真办理人大代表建议和政协提案。“五五”普法通过验收。行政审批制度改革继续深化，政府法制工作得到加强。我市“宝贝回家”志愿者协会张宝艳获得“12·4中国十年法治人物”殊荣。创建了“N+1”矛盾纠纷排查调处模式，加强社会治安综合治理，深入开展严打斗争，平安通化建设取得新成效。全面开展安全生产隐患排查治理，在重点行业和领域设立岗位监督员，各类事故和人员伤亡实现双下降。食品药品安全监管工作得到加强。加大信访工作力度，解决了一批信访突出问题。市长公开电话受理群众来电5.1万件，办复率99%。妥善预防和处置突发事件。组织开展了第六次人口普查。认真开展“创先争优”活动和“解放思想、改革创新、转变方式、科学发展”主题教育，深入推进精细化管理和“查找改”。制定出台行政问责若干规定，强化了行政责任和绩效评估。深化政行风民主评议和纠风专项治理，软环境进一步改善，反腐倡廉建设取得新成效。省委、省政府先后在我市召开了新农保、“暖房子”工程、农业产业化、信访事项核查认定终结试点等现场会和经验交流会。

　　各位代表！五年来取得的成就极为不易，这是市委正确领导的结果，是市人大、市政协监督支持的结果，是全市人民团结奋斗的结果，是社会各界共同参与的结果。在此，我代表市政府，向辛勤工作在各行各业的全市人民，向给予政府工作大力支持的人大代表和政协委员，向各民主党派、工商联、无党派人士和人民团体，向驻通国省属单位、驻通部队和武警官兵，向所有关心支持通化发展的同志们、朋友们，表示衷心的感谢！

　　我们也清醒地看到存在的问题，主要是经济发展后劲还不强，大项目、大企业不多；经济结构不尽合理，经济规模和增长质量有待提高，自主创新能力不强，转变粗放型经济增长方式任重道远；城镇化发展基础薄弱，中心城市规模小，尚未形成较强的辐射带动功能；富民进程推进不快，城乡居民收入增长较慢，一些群众的利益诉求还没有得到很好解决；影响社会和谐稳定的因素大量存在，社会管理亟待创新，维护社会稳定的任务十分繁重。与此同时，还存在部分公职人员责任意识不强、精神状态不佳、工作作风不实等问题。我们一定高度重视，采取有力措施，认真加以解决。

　　二、“十二五”发展目标和主要任务

　　“十二五”是全面建设小康社会的关键时期，是深化改革开放、加快转变经济发展方式的攻坚阶段。面对“不进则退、小进也是退”的现实和人民群众日益增长的物质文化需求，我们要牢牢把握“发展”和“民生”两个关键，深刻认识发展的决定性意义，励精图治，奋发图强，坚定不移地快发展、大发展，确保经济增长指标和民生发展指标高于全省平均水平。

　　“十二五”时期，我市经济社会发展的指导思想是：坚持以科学发展观为指导，深入落实党的十七届五中全会、省委九届十一次全会和市委五届八次全会精神，以解放思想、改革创新、转变方式、科学发展、加快振兴、富民强市为主题，统筹推进具有通化特色的工业化、城镇化、农业现代化，着力实施投资拉动、项目带动和创新驱动战略，以增量带动结构优化，以创新促进产业升级，以发展保障民生改善，强力推动资源产业化、产业规模化、生产低碳化、交通快速化、人居生态化，大力弘扬“真诚包容、勇于担当、重行务实、创业自强”的城市精神，建设东北东部地区中心城市和中国北方宜居山水城市，加快实现通化老工业基地全面振兴，努力让城乡居民生活得更加美好，为全面建设小康社会奠定更加坚实的基础。

　　“十二五”的主要奋斗目标：地区生产总值达到1500亿元，年均增长20%。人均GDP达到64377元；全口径财政收入突破150亿元，年均增长20%，其中地方级财政收入达到66.4亿元，年均增长15%；规模以上工业总产值突破2000亿元，年均增长20%以上；社会消费品零售总额达到570亿元，年均增长18%；城镇居民人均可支配收入达到32154元，年均增长14%；农民人均纯收入达到11334元，年均增长13%。

　　实现上述目标，必须坚持发展是硬道理不动摇，推动经济发展方式加快转变，促进科学发展、快速发展；必须坚持改革开放不动摇，进一步加大开放力度，注重以开放促改革；必须坚持工业立市、产业兴市不动摇，加快建设新型工业基地、农特产业强市、区域性服务中心；必须坚持做大中心城市不动摇，统筹推进具有通化特色的城镇化；必须坚持发展为民、推进全民创业不动摇，把改革发展成果惠及百姓，以创业带动民富。

　　（一）保持投资规模适度增长，在重点项目建设上取得突破。加大工业项目、技术改造、高新技术、基础产业、基础设施、民生和社会事业等投资，固定资产投资年均增长20%以上，工业投资占全部投资的比重超过50%。开工建设亿元以上项目830项，滚动实施10亿元以上重大项目100项。扶持骨干企业和现有工业项目释放产能，新培育60户产值超亿元的大企业。坚持招商引资与合作重组并举，引进100个投资5亿元以上的大项目。围绕扩能改造、提高配套率、前移后延产业链、增强自主创新能力、发展资源特色产业、城镇化建设和民生改善，谋划储备亿元以上项目1000项。

　　（二）推动产业升级，在新型产业基地建设上取得突破。抓好医药、冶金、食品、能源、机械制造、建材、化工、信息八大产业跃升，促进工业化与信息化融合。建设国家新型工业化医药产业示范基地，推动医药工业、医药商贸、医药科研、医药教育、中药材基地、医药康复、医药文化协调发展，整体塑造“通化医药”品牌，建成国际化程度较高的“医药名城”。建设“精品钢城”和千万吨级新型钢铁基地，支持通钢完成装备大型化改造，基本建成通钢新区，积极发展原辅材料配套、板材深加工、型材制品化等兴钢产业，提高钢材就地转化率和制品化率，兴钢产业形成集群效应。建设“绿色食品基地”，加强长白山特色食品资源系列开发，发展天然绿色食品加工业，培育壮大系列品牌。建设“中国通化葡萄酒城”，把葡萄酒产业规模做大、质量做优、市场做活、品牌做响，提升“通化葡萄酒”的知名度和美誉度。到2015年，医药产业产值达到1000亿元，冶金产业产值达到600亿元，食品产业产值达到350亿元。大力发展高新技术产业，培育发展生物制药、新材料、新能源、节能环保、高端装备制造等战略性新兴产业，扶持金属镁及深加工产业快速发展，发展汽车零部件、医疗器械等接续替代产业。深入实施品牌战略，新创一批中国名牌产品、地理标志保护品种和驰名商标。建设高品位旅游项目、高档次服务设施，打造区域性观光休闲度假中心和中远程旅游目的地。推动现代物流、商贸、文化等服务业跨越发展，发展旅游文化商贸复合型支柱产业，将通化建设成为东北东部物流中心、商贸中心和长白山地域文化中心。服务业增加值年均增长20%以上。

　　（三）统筹城乡发展，在城镇化建设上取得突破。按照“两轴、两区、一带”格局，高起点搞好城镇规划，注重强化中心、构建支点、区域联动、城乡互动，形成具有通化特色的城镇发展体系。推进通化都市区率先突破，加快旧城改造、新区开发、新城建设，完善市政设施和综合交通网络，拓展城市发展空间，增强中心城市的吸引力、带动力。围绕“一主二辅四组团”和通快城市发展轴，实行市县协调同步规划建设，实施一批彰显特色、提高品位、惠及民生的重点工程。打造“通梅经济走廊”，发展“通梅集城镇发展轴”，建设产业集聚走廊、城乡安居走廊。支持梅河口依托重要节点城市的优势，建设“梅柳辉经济协作区”中心城市。打造鸭绿江旅游生态发展带，抓好集安旅游城市建设。加快县城扩容发展和城关镇、中心镇建设，完善综合功能、提高承载能力。创新城市管理模式，推行精细化管理、人性化服务，促进人与城市和谐发展。加强农村水、电、路、气等设施建设和环境整治，整体提升新农村建设水平。到2015年，通化都市区人口发展到70万，经济强镇达到50个，省级示范达标村达到215个，全市城镇化率达到60%以上。

　　（四）构建现代农业产业体系，在农特产业强市建设上取得突破。深入推进农、林、牧、特资源转化，加快发展富有通化特色的现代农业。实施绿优米等优质粮工程，建好生态、节水、农机、科技4个农业示范区，提高农业综合生产能力。推动畜牧业扩能升级，建设1200个标准化牧业小区。加快特产业发展，推进参、药、蛙、蜂等特色资源深度开发。拓展人参研发加工领域，积极开发药食同源新产品，促进人参产业振兴，建设集科研、种植、加工、商贸、旅游、文化于一体的“中国人参产业基地”。大力发展林业经济和家具制造业。发展山葡萄种植基地，建立山葡萄酒标准体系。实施棚膜蔬菜工程，建设以东昌区、二道江区为主的周边产业区和县（市）及公铁沿线蔬菜产业带，逐步提高城市蔬菜自给能力。抓好农产品品牌培育、科技创新和市场开发。健全动植物疫病防控、农产品质量安全检疫检测体系。到2015年，畜牧业产值达到150亿元，园艺特产业产值达到400亿元，人参产业产值达到180亿元，林业产值达到158亿元，农产品加工转化能力达到280万吨。

　　（五）推进基础设施重点工程，在开放通道和保障能力建设上取得突破。加快建设连接腹地、通边出海的高速公路、快速铁路、机场、陆港和口岸，建成综合交通网络和东北东部区域交通中心。高速公路通车里程达到525公里，公路总里程突破7000公里。规划建设水利工程383项，基本形成防洪、供水、灌溉等保障体系。新建扩建5座220千伏变电站、28座66千伏变电站，新增变电容量70万千伏安。加快抽水蓄能、油母页岩、生物发电等项目建设，加强铁矿、煤炭等资源勘探开发，发展沼气、太阳能等清洁能源。推动“气化通化”，气化率由16%提高到62%。实施“数字通化”工程。

　　（六）深化改革开放，在体制机制创新上取得突破。统筹推进户籍、土地等城镇化综合配套改革，推动城乡社会保障体系衔接。支持企业引进战略投资者，推进跨行业、跨区域合作重组。深化财税体制改革，健全公共财政体系。创新金融服务，扩大资本市场融资和信贷投放规模，争取建立区域股权交易中心。稳步推行厂办大集体企业改革。抓好资源要素配置市场化改革。推进科教文卫等事业单位分类改革，提高社会事业发展活力。优化民营经济发展环境，大力推动全民创业，民营经济占经济总量的比重达到80%以上，对财政的贡献率达到70%以上。拓展对外开放的广度和深度，对接长吉图，融入环渤海，把通化建成东北东部区域的近海地区、前沿门户。推动通化经济开发区、7个县（市、区）开发区及各类特色园区加速发展。加大招商引资力度，多引进大企业、大项目。扶持外贸骨干企业和出口基地发展，扩大对外贸易。引进内资年均增长20%，实际利用外资和外贸进出口总额年均分别增长15%。

　　（七）实施科教兴市和人才战略，在社会事业发展上取得突破。加快自主创新和引进消化吸收再创新，基本建成以企业为主体、产学研紧密结合的技术创新体系。新发展企业技术中心30家以上，高新技术产业增加值占GDP的比重达到15%，科技进步贡献率超过全省平均水平。完善知识产权创新体系。坚持教育优先发展，促进教育均衡发展，推动教育公平发展，提高教育质量和水平。完成中小学标准化建设。发展职业教育，培育大型职教集团和实训基地。以通化师院为基础，筹划建设通化大学。积极发展幼教事业。确立人才优先发展战略布局，统筹人才队伍建设，培养和引进一批技能型、创业型、创新型人才。繁荣发展文化事业和文化产业，培育地域文化中心、知名文化品牌，形成覆盖城乡的公共文化服务体系。规划建设文化产业园区、主题文化公园等重点项目，催生新的文化业态。发展群众体育运动，完善体育基础设施。加强基本医疗保障制度建设，促进基本公共卫生服务均等化，提高医疗救治水平和传染病防控能力。坚持计划生育基本国策，统筹解决人口问题。推动老龄事业加快发展。推进社会管理创新，加强和改进群众工作。健全信访终结机制，强化安全生产管理监督，深化严打斗争，创建平安通化，保持社会和谐稳定。实施“强基富民固边”工程，维护边境安全稳定发展。广泛开展精神文明创建活动，争创全国文明城市。

　　（八）加强生态建设和环境保护，在可持续发展上取得突破。推行资源节约、环境友好的生产方式和消费方式，保护通化特色生态资源。实施一批节能减排重点工程，发展低碳经济、循环经济。争取进入国家资源型城市可持续发展试点行列。抓好污染治理和自然灾害防治，强化饮用水源、湿地、土地、森林、矿产等资源的生态保护。建成哈泥河功能保护区。继续开展城乡环境综合整治，创建国家卫生城市、园林城市和环保模范城市。万元GDP能耗下降15%，化学需氧量、二氧化硫排放量分别下降10%和5%，工业固体废弃物综合利用率达到90%，森林覆盖率达到64.7%，城市饮用水源水质达标率超过95%，生活污水、垃圾无害化处理率分别达到80%和85%。

　　（九）实施富民工程，在保障和改善民生上取得突破。大力发展有利于群众增收的产业和企业，扩大财富源泉和创造财富人群比重，强化发展对富民的支撑能力。实施更加积极的就业政策，建立覆盖城乡的社会保障体系，稳步提高居民收入。合理调整收入分配关系，促进社会工资较快增长，增加居民财产性收入。提高农民职业技能和创收能力，拓宽农民增收渠道。扩大住房公积金制度覆盖面，推进住房保障体系建设。加快实施保障性住房工程和“暖房子”工程，让城乡居民住有所居、住得温暖。完成“六路安居”工程600万平方米，改善18万户居民住房条件。加强社会救助体系建设，扩大助学、助困、助医、助孤、助老、助残范围，保障困难群众基本生活。

　　三、2011年工作安排

　　2011年是实施“十二五”规划的第一年，我们要踏石留印、抓铁有痕，确保各项工作高标准起步、高起点开局。预期地区生产总值增长20%；全口径财政收入增长22.6%，其中地方级财政收入增长19.4%；固定资产投资增长30%以上；城镇居民人均可支配收入、农民人均纯收入分别增长13%和11%。

　　（一）毫不动摇抓项目、抓招商。紧紧把握国家政策取向，优化投资结构。新建、续建3000万元以上项目750项，亿元以上项目240项，10亿元以上项目24项。开工建设通钢120万吨回转窑、120吨转炉、2680立方米高炉、60万吨精品棒材项目，加快东宝人胰岛素及系列生物制品、大容量注射剂和双龙化工产业园、华能热电、安柏生物可得然胶、科泰减振复合钢板等项目实施进度，确保按计划竣工投产。整合各方面资源，建立完善相应工作机制，谋划重大项目。加强项目融资，完善投融资平台运行机制，争取金融机构和政策性银行信贷投放。创新招商方式，实行多线推进、分工协作，紧盯央企、突出民企、注重外企，引进一批基地型、龙头型项目。引进亿元以上项目320项，实际利用外资1.85亿美元。通化经济开发区新招、开工和投产亿元以上项目30项。推动7个县（市、区）开发区提档升级。加快通丹经济带建设，完善跨区域合作机制，协同推进各项产业建设，积极开展合作招商。

　　（二）千方百计促进工业提速增效。深入实施产业跃升计划，发展壮大8个特色工业园区、6个省级产业集群，加强银企对接、要素协调和工业生产运行组织，新口径规模以上工业产值增长20%。加快省级医药高新技术产业开发区建设，做好国家级开发区申报工作。申请注册“通化医药”地理标志证明商标。做好通钢新区规划和开工建设前期工作，全面建设“五鸭铁”兴钢新区。规划建设金厂镁合金新区，支持深加工企业加快发展。做好“葡萄酒城”总体规划，在企业整合重组及市场开发、规范管理和建设葡萄酒酒庄等方面寻求突破。制定出台战略性新兴产业政策，促进产业拉长链条。抓好工业“五个一批”工程。完成技改投资200亿元。支持企业盘活存量、释放产能。深入开展质量兴市活动，培育具有竞争优势的名牌产品。加强10户重点企业节能监测，支持节能环保、低碳能源技术开发和企业节能改造，完成省下达的节能减排指标任务。开展“中小企业服务年”活动，实施“万名创业者”、“万名小老板”培育工程，建设8个省级中小企业孵化基地，推动民营经济腾飞。民营经济主营业务收入增长20%，实缴税金增长18%。

　　（三）持之以恒发展农业农村经济。调整优化种植结构，推动粮食增产增效。抓好20个千万元以上产业化项目，启动建设国家级农副产品精深加工产业园、肥肝系列产品生产线等项目，支持卓越、正方、中兴、阜康等龙头企业加快发展。实施畜牧业发展攻坚，强化动物疫病防控，建设2个省级牧业产业园区，新建130个标准化牧业小区，畜牧业产值增长22%。实施特产业重点项目12项，园艺特产业产值增长15%。抓好人参种植规模化、质量标准化、加工精深化、产业集约化，人参加工量突破4000吨。建成长白山人参贸易区。启动第二个十年绿化美化吉林大地活动。发展林业经济和林木产品加工业，造林绿化10万亩。完成集体林权配套改革。制定城市“菜篮子”建设发展规划，扩大蔬菜种植面积，推进蔬菜标准化生产。蔬菜种植超过30万亩，棚膜蔬菜达到6万亩。抓好“百村示范、全面提升”工程、“清洁文明提升”工程和“民俗特色旅游示范村建设”工程，实施一批富民项目。开展农民技能培训和创业指导，县域务工农民达到16万人。新建通屯公路450公里。落实农村“三项制度”，高标准完成乡（镇）“三资”代理服务中心建设。加快发展县域经济，辐射带动农村各业发展。

　　（四）下大力气推动服务业跨越发展。加快建设长白山医药物流中心、二密物流仓储基地和三源浦空港经济区，筹建东北东部土特产品、通化成品粮油、梅河大米等区域批发市场，合理规划布局蔬菜批发市场、农贸市场、社区菜店。建设义乌国际小商品配货中心、高力休闲餐饮文化城、戴斯五星级酒店等项目，推动江南商贸新城扩大规模、提高层次。启动湾湾川生态新城规划建设，加快打造通快同城化发展连接带。实施旅游业壮大计划，抓好滑雪度假园区、旅游综合服务中心、朝鲜族民俗园等45个旅游项目，强化宣传推介和客源市场及商品开发，创建中国优秀旅游目的地，旅游收入增长20%。实施文化产业提速计划，挖掘、整合地域特色文化资源，建立文化产业融资服务平台，培育文化产业龙头企业。做大做精松花石（砚）产业和关东文化产品交易市场。积极引进股份制银行，支持企业上市融资。改善担保结构，增强融资能力。规范房地产市场开发，满足不同收入家庭住房需求。完善城市商业功能，继续抓好“万村千乡市场工程”，发展新型消费业态。社会消费品零售总额增长19%。加强医药、钢铁、农特产品出口基地建设，扩大出口规模，实现外贸进出口总额7亿美元。

　　（五）进一步加快基础设施建设。重点抓好十项工程：一是高速公路工程。通丹、梅沈高速公路建成通车，全线推进通靖高速公路建设，完成和抓好通长、通集高速公路前期工作。二是铁路工程。通丹铁路竣工通车，争取开工建设四平至通化至松江河快速铁路、沈吉铁路复线，筹划通集快速铁路。三是机场工程。启动建设飞行区、航站区、候机楼和配套设施工程，力争年内竣工。四是陆港工程。建设保税库、海关联检大楼、集装箱场站和铁路专用线，一期工程投入使用。五是电力工程。新建扩建66千伏线路19条、变电站17座。加快中朝电站项目建设。六是水利工程。实施浑江干流整治、水库除险加固、水土流失治理和灌区配套及节水改造等50项工程。七是生态环保工程。实施市区垃圾焚烧发电、垃圾处理厂改造和通钢烧结脱硫工程，医疗废弃物处理、哈泥河人工湿地污水处理和集安市、通化县污水处理及梅河口市、柳河县垃圾处理工程建成使用。加强城区周山绿化和环境综合整治。八是城市公共设施工程。继续推进地下管网改造、架空线路落地和市政设施功能提升，规划建设市区备用水源。九是土地开发整理工程。复垦耕地5.1万亩，开发整理土地5328亩。十是信息产业工程。加快“数字通化”项目建设，启动无线数字一期工程。

　　（六）加大城市建设与管理力度。编制完成都市区控制性详规和专项规划，建设城市规划馆。市区城建计划投资37亿元。拆迁棚户区和危旧房100万平方米，开工建筑面积150万平方米。继续推进“暖房子”工程，综合整治旧住宅小区300栋，完成既有建筑节能改造100万平方米。对滨江东路盛和药业至金厂河口段进行改造，修建江堤和人行道，延伸沿江景观带。实施精品街建设工程，改造维修50条主次街路巷路和二道江区东学路。新建续建江南新区5条路网和水热气等基础设施，加快江南商贸新城开发建设。改造供水供热供气设施，解决高区及周边1万人用水、4000户居民用气问题，改造供热管网48公里，实施分户供热、热计量安装100万平方米。综合改造城市西出口和北出口，打造特色景观。实施绿化美化工程，栽植乔木、灌木、花草100万株。推进滨江沿河、街路巷路亮化升级和楼道照明设施改造。治理截洪沟、光复河，提高防洪排涝能力，改善沿河环境。坚持建管并重，组织开展“城市管理年”活动，加强环境卫生、市容市貌、市政设施和物业管理，健全城市管理长效机制。争创国家卫生城市，启动国家环保模范城市创建工作。

　　（七）坚定不移改善民生、维护稳定。实施2011年民生工作计划，集中力量办100件左右民生实事。抓好就业服务平台建设，推进就业困难人员、高校毕业生等群体就业，城镇新增就业5万人。提高基本养老金、失业保险金水平和城乡低保对象生活补助标准，实施医疗、工伤、生育保险市级统筹。扩大新型农村养老保险试点范围，做好五七家属工参保工作。完善社会救助体系，保障好困难群体的生活。建成廉租房2万平方米，为1.7万户低收入家庭发放住房租赁补贴。改造农村危旧房7000户。新建农村饮水安全工程205处。继续抓好灾后重建，239项水毁水利工程汛期前完成修复，基本完成道路交通、公共服务设施和水毁耕地恢复整治等重建任务。强化安全生产监督管理，超前排查整改安全隐患，坚决遏制重特大事故发生。加强公共安全设施、安全生产和消防设施建设，抓好防灾救灾体系建设和全民安全教育，提升安全保障能力。推进社会治安防控体系建设，严厉打击各类犯罪，增强人民群众的安全感。认真做好信访工作，及时解决群众诉求。加强应急管理，强化社会稳定风险评估，有效预防和排查调处矛盾纠纷，积极推进社会治安综合治理和平安通化建设。加强价格监测监管，合理使用价调基金，建立健全社会救助标准与物价上涨挂钩联动机制，依法打击恶意囤积、哄抬物价等违法行为，保障市场供应和价格总水平基本稳定。深入开展食品药品安全专项整治，让人民群众吃的放心、用的安全。

　　（八）协调发展各项社会事业。实施科技创新“310”工程，申报国家和省科技项目65项、重大专项15项，申请专利240件。加强学前教育，推进素质教育。改造维修校舍18万平方米，全面完成校舍安全工程。抓好职业教育校企合作、集团化办学，发挥职教园区作用。市青少年宫全面开放。深化医药卫生体制改革，推进基本药物制度实施，加快公共卫生服务项目建设，稳步提高基本医疗保障水平，做好重大传染病疫情防控工作。抓好文化惠民工程，打造文艺精品。完成包括“五馆”在内的科技文化活动中心主体工程，做好文物征集和布展筹划。争取启动档案馆建设。实施自安山城保护性抢救工程，规划建设王八脖子考古遗址公园。开展全民健身活动，举办首届全市运动会，备战第十七届省运会。抓好市县乡村四级公共文体设施建设。扩大广播电视户户通工程覆盖面。抓好优生优育促进工作，人口自然增长率控制在3.1‰以内。完善社区管理和服务功能。关心妇女、儿童、老龄、残疾人事业。推动民族团结进步和民族村镇加快发展。加强国防教育，搞好双拥共建和国防后备力量建设，争创全国双拥模范城“七连冠”。

　　（九）努力建设人民满意政府。从市政府领导班子和组成人员做起，坚持“科学理论武装、具有世界眼光、善于把握规律、富有创新精神”，立足当前，着眼长远，正确处理整体与局部、重点与一般的关系，统筹推进全局工作。主动接受人大法律监督、工作监督和政协民主监督以及人民群众和新闻舆论监督，强化决策咨询，发扬民主、集思广益，提高决策的科学化、民主化水平。把依法行政贯穿于政府工作全过程，落实行政执法责任制，规范执法、文明执法、公正执法，维护社会公平正义。继续清理行政审批事项和非行政许可项目，简化审批流程。用硬措施优化软环境，推进政务公开，为企业和投资者提供“一如三优三全”服务。推行绩效评估，严格行政问责，推动责任链接无缝化。转变作风，干部在一线指挥、工作在一线落实、问题在一线解决。聚焦发展，倾注民生，为群众诚心诚意办实事，尽心竭力解难事，坚持不懈做好事。妥善解决群众关心的热点难点问题，努力做到“件件有着落、事事有回音”。坚持律己律人律事相统一，狠抓反腐倡廉，加强行政监察、审计监督，严肃查处违法违纪案件。全面加强政府机关建设，使广大公职人员增强大局意识、责任意识、忧患意识，立足本职，干好每一天、做好每件事。

　　各位代表！实现“十二五”的宏伟目标，是全市人民的热切期盼，是时代赋予我们的光荣使命。让我们在市委的正确领导下，在市人大、市政协的大力支持下，凝聚全市人民的智慧和力量，坚韧不拔，攻坚克难，只争朝夕，真抓实干，续写通化老工业基地振兴崛起、城乡居民生活更加美好的壮美篇章！
